# Restoring Color in Woolen or Cotton Fabrics

**Published:** 1883-05

**Authors:** 


Restoring Color in Woolen or Cotton Fabrics.—When color on a fabric has been accidentally or otherwise destroyed by acid, ammonia is applied to neutralize the same, after which an application of chloroform will, in almost all cases, restore the original color. The application of ammonia is common but that of chloroform is but little known.

